# A unified catalog of 204,938 reference genomes from the human gut microbiome

**DOI:** 10.1038/s41587-020-0603-3

**Published:** 2020-07-20

**Authors:** Alexandre Almeida, Stephen Nayfach, Miguel Boland, Francesco Strozzi, Martin Beracochea, Zhou Jason Shi, Katherine S. Pollard, Ekaterina Sakharova, Donovan H. Parks, Philip Hugenholtz, Nicola Segata, Nikos C. Kyrpides, Robert D. Finn

**Affiliations:** 1 European Bioinformatics Institute (EMBL–EBI), Wellcome Genome Campus, Hinxton, UK; 2 Wellcome Sanger Institute, Wellcome Genome Campus, Hinxton, UK; 3 US Department of Energy Joint Genome Institute, Walnut Creek, CA USA; 4 Environmental Genomics and Systems Biology Division, Lawrence Berkeley National Laboratory, Berkeley, CA USA; 5 Enterome Bioscience, Paris, France; 6 Gladstone Institutes, San Francisco, CA USA; 7 Chan Zuckerberg Biohub, San Francisco, CA USA; 8 Institute for Human Genetics, University of California San Francisco, San Francisco, CA USA; 9 Institute for Computational Health Sciences, University of California San Francisco, San Francisco, CA USA; 10 Quantitative Biology Institute, University of California San Francisco, San Francisco, CA USA; 11 Department of Epidemiology and Biostatistics, University of California San Francisco, San Francisco, CA USA; 12 Australian Centre for Ecogenomics, School of Chemistry and Molecular Biosciences, The University of Queensland, Brisbane, Queensland Australia; 13 CIBIO Department, University of Trento, Trento, Italy

**Keywords:** Microbiome, Metagenomics

## Abstract

Comprehensive, high-quality reference genomes are required for functional characterization and taxonomic assignment of the human gut microbiota. We present the Unified Human Gastrointestinal Genome (UHGG) collection, comprising 204,938 nonredundant genomes from 4,644 gut prokaryotes. These genomes encode >170 million protein sequences, which we collated in the Unified Human Gastrointestinal Protein (UHGP) catalog. The UHGP more than doubles the number of gut proteins in comparison to those present in the Integrated Gene Catalog. More than 70% of the UHGG species lack cultured representatives, and 40% of the UHGP lack functional annotations. Intraspecies genomic variation analyses revealed a large reservoir of accessory genes and single-nucleotide variants, many of which are specific to individual human populations. The UHGG and UHGP collections will enable studies linking genotypes to phenotypes in the human gut microbiome.

## Abstract (web summary)

More than 200,000 gut prokaryotic reference genomes and the proteins they encode are collated, providing comprehensive resources for microbiome researchers.

## Main

The human gut microbiome has been implicated in important phenotypes related to human health and disease^1,2^. However, incomplete reference data that lack sufficient microbial diversity^3^ hamper understanding of the roles of individual microbiome species and their functions and interactions. Hence, establishing a comprehensive collection of microbial reference genomes and genes is an important step for accurate characterization of the taxonomic and functional repertoire of the intestinal microbial ecosystem.

The Human Microbiome Project (HMP)^4^ was a pioneering initiative to enrich knowledge of human-associated microbiota diversity. Hundreds of genomes from bacterial species with no sequenced representatives were obtained as part of this project, allowing their use for the first time in reference-based metagenomic studies. The Integrated Gene Catalog (IGC)^5^ was subsequently created, combining the sequence data available from the HMP and the Metagenomics of the Human Intestinal Tract (MetaHIT)^6^ consortium. This gene catalog has been applied successfully to the study of microbiome associations in different clinical contexts^7^, revealing microbial composition signatures linked to type 2 diabetes^8^, obesity^9^ and other diseases^10^. But, as the IGC comprises genes with no direct link to their genome of origin, it lacks contextual data to perform high-resolution taxonomic classification, establish genetic linkage and deduce complete functional pathways on a genomic basis.

Culturing studies have continued to unveil new insights into the biology of human gut communities^11,12^ and are essential for applications in research and biotechnology. However, the advent of high-throughput sequencing and new metagenomic analysis methods—namely, involving genome assembly and binning—has transformed understanding of the microbiome composition in both humans and other environments^13–15^. Metagenomic analyses are able to capture substantial microbial diversity not easily accessible by cultivation by directly analyzing the sample genetic material without the need for culturing, although biases do exist^16^. This can be achieved by binning de novo-assembled contigs into putative genomes, referred to as metagenome-assembled genomes (MAGs). However, current challenges associated with metagenome assembly and binning can result in incorrectly binned contigs, which substantially affects further taxonomic and functional inferences. Therefore, the use of MAGs requires careful considerations^17^, but they provide important insights into the uncultured microbial diversity in the absence of isolate genomes.

Recent studies have massively expanded the known species repertoire of the human gut, making available unprecedented numbers of new cultured and uncultured genomes^16,18–21^. Two culturing efforts isolated and sequenced over 500 human-gut-associated bacterial genomes each^19,21^, while three independent studies^16,18,20^ reconstructed 60,000–150,000 MAGs from public human microbiome data, most of which belong to species lacking cultured representatives. Combining these individual efforts and establishing a unified nonredundant dataset of human gut genomes is essential for driving future microbiome studies. To accomplish this, we compiled and analyzed 204,938 genomes and 170,602,708 genes from human gut microbiome datasets to generate the Unified Human Gastrointestinal Genome (UHGG) and Protein (UHGP) catalogs, the most comprehensive sequence resources of the human gut microbiome established thus far.

## Results

### More than 200,000 human gut microbial genomes in the UHGG catalog

**Fig. 1:**
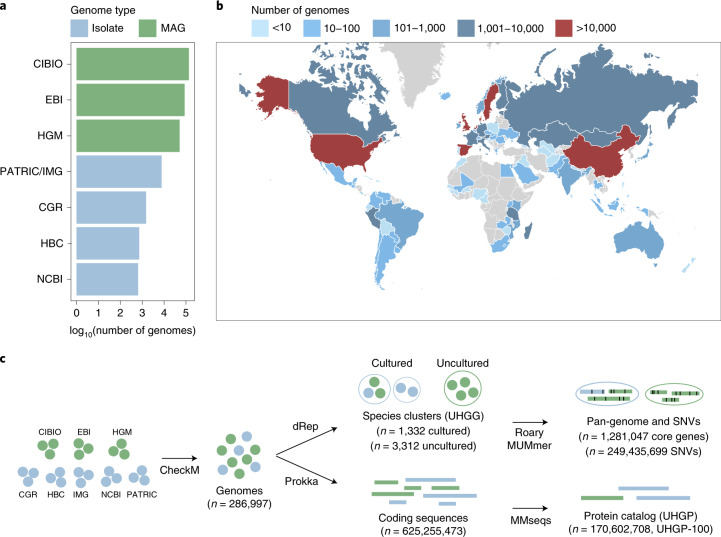
The unified sequence catalog of the human gut microbiome. **a**, Number of gut genomes for each study set used to generate the sequence catalogs, colored according to whether they represent isolate genomes or MAGs. **b**, Geographic distribution of the number of genomes retrieved per country. **c**, Overview of the methods used to generate the genome (UHGG) and protein sequence (UHGP) catalogs. Genomes retrieved from public datasets first underwent quality control by CheckM. Filtered genomes were clustered at an estimated species level (95% ANI), and their intraspecies diversity was assessed (genes from conspecific genomes were clustered at 90% protein identity). In parallel, a nonredundant protein catalog was generated from all coding sequences of the 286,997 genomes at 100% (UHGP-100, *n* = 170,602,708), 95% (UHGP-95, *n* = 20,239,340), 90% (UHGP-90, *n* = 13,907,849) and 50% (UHGP-50, *n* = 4,735,546) protein identity.

We first gathered all prokaryotic isolate genomes and MAGs from the human gut microbiome (publicly available as of March 2019). We compiled the isolate genomes from the Human Gastrointestinal Bacteria Culture Collection (HBC)^19^ and the Culturable Genome Reference (CGR)^21^, as well as cultured human gut genomes available in the NCBI^22^, PATRIC^23^ and IMG^24^ repositories, which include genomes from several other large studies^11,12,25^. In addition, we included all of the gut MAGs generated in Pasolli et al.^20^ (CIBIO), Almeida et al.^18^ (EBI) and Nayfach et al.^16^ (HGM). To standardize the genome quality across all sets, we used thresholds of >50% genome completeness and <5% contamination, combined with an estimated quality score (completeness –5 × contamination) > 50. The final numbers of genomes matching these criteria were 734 (HBC), 1,519 (CGR), 651 (NCBI), 7,744 (PATRIC/IMG), 137,474 (CIBIO), 87,386 (EBI) and 51,489 (HGM), resulting in a total of 286,997 genome sequences (Fig. 1a and Supplementary Table 1). These represented 204,938 nonredundant genomes on the basis of a Mash^26^ distance threshold of 0.001 (99.9% nucleotide identity) and only considering one genome per species per sample to account for the fact that the three large MAG studies analyzed many samples in common. Genomes were recovered in samples from a total of 31 countries across six continents (Africa, Asia, Europe, North America, South America and Oceania), but the majority originated from samples collected in China, Denmark, Spain and the United States (Fig. 1b).

**Extended Data Fig. 1:**
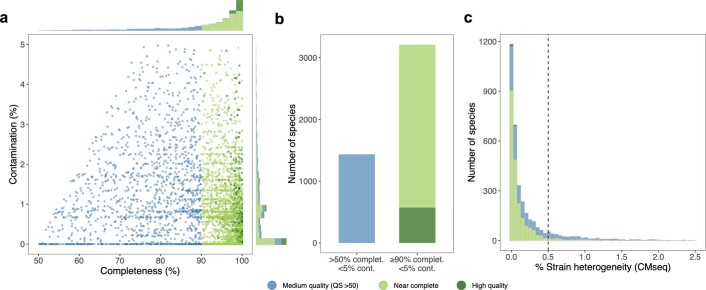
Genome quality of species representatives. **a**, Completeness and contamination scores for each of the 4,644 species representatives, colored by their quality classification category. Medium quality: >50% completeness; near complete: ≥90% completeness; high-quality: >90% completeness, presence of 5S, 16S and 23S rRNA genes, as well as at least 18 tRNAs. All genomes have a quality score (QS = completeness – 5 × contamination) above 50. **b**, Number of species according to different completeness and contamination criteria. **c**, Distribution of the level of strain heterogeneity (proportion of non-synonymous substitutions) estimated for the species-level MAGs using CMseq. Dashed vertical line corresponds to the threshold defined in Pasolli, et al.^20^ to distinguish medium- from high-quality MAGs.

**Extended Data Fig. 2:**
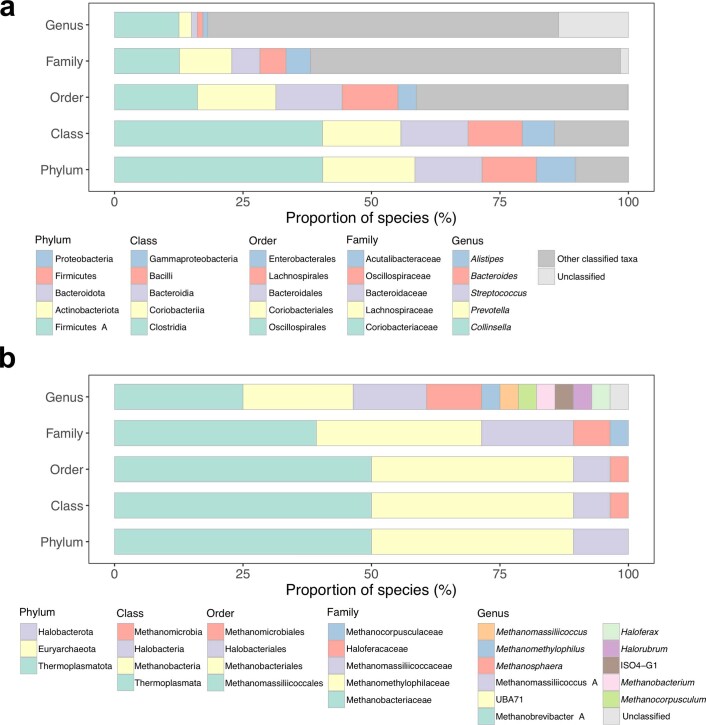
Taxonomy composition of the bacterial and archaeal species. **a**, Taxonomic affiliation of the 4,616 bacterial species detected. Data is partitioned by taxonomic rank, with only the five most highly represented taxa per rank depicted in the legend. **b**, Taxonomic affiliation of the 28 archaeal species detected, partitioned by taxonomic rank.

To determine how many species were included in this gut reference collection, we clustered all 286,997 genomes using a multistep distance-based approach (Methods) with an average nucleotide identity (ANI) threshold of 95% over at least a 30% alignment fraction (AF)^27^. The clustering procedure resulted in a total of 4,644 inferred prokaryotic species (4,616 bacterial and 28 archaeal; Supplementary Table 2). We found the species clustering results to be highly consistent with those previously obtained^16,18,20^ (Supplementary Table 3). The best quality genome from each species cluster was selected as its representative on the basis of genome completeness, minimal contamination and assembly N50 (with isolate genomes always given preference over MAGs), and the final set was used to generate the UHGG catalog (Fig. 1c). Of the 4,644 species-level genomes, 3,207 were >90% complete (interquartile range, IQR = 87.2–98.8%) and <5% contaminated (IQR = 0.0–1.34%), with 573 of these having the 5S, 16S and 23S rRNA genes together with at least 18 of the standard tRNAs (Extended Data Fig. 1). These 573 genomes (535 from isolates and 38 from MAGs) satisfy the ‘high quality’ criteria set for MAGs by the Genomic Standards Consortium^28^. The rRNA operon has previously been shown to be a problematic region to assemble from short-read metagenomic datasets^13,16,18,20^, which might explain the low number of high-quality MAGs. Thereafter, we classified each species representative using the Genome Taxonomy Database Toolkit^29,30^ (GTDB-Tk; Extended Data Fig. 2), a standardized taxonomic framework based on a concatenated protein phylogeny representing >140,000 public prokaryote genomes, fully resolved to the species level (see Methods for details on the taxonomy nomenclature used). However, over 60% of the gut genomes could not be assigned to an existing species, confirming that the majority of the UHGG species lack representation in current reference databases.

To obtain further insights into the quality of the UHGG genomes, we inferred the level of strain heterogeneity within each MAG with CMseq^20^. The median strain heterogeneity (proportion of polymorphic positions) of the UHGG MAGs was 0.06% (IQR = 0.01–0.25%; Extended Data Fig. 1c and Supplementary Table 1), which is below the 0.5% threshold defined previously^20^ to distinguish medium- from high-quality MAGs. We believe that this additional metric on strain heterogeneity is a useful complement to the standard completeness and contamination estimates, providing further evidence of the overall high quality of the genomes included here.

### Comparison of species recovered in individual studies

**Fig. 2:**
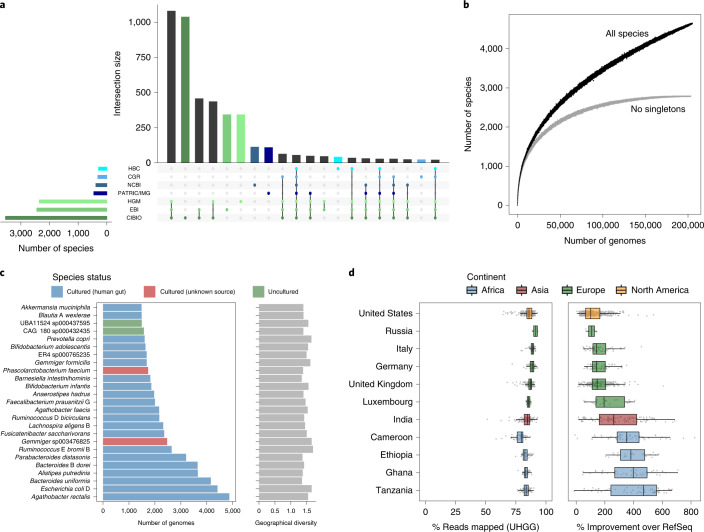
Intersection and frequency of species across studies. **a**, Number of species found across genome study sets, ordered by their level of overlap. Vertical bars represent the number of species shared between the specific study sets highlighted with colored dots in the lower panel. Horizontal bars in the lower panel indicate the total number of species contained in each study set. Different shades of green denote the study sets represented exclusively by MAGs, whereas those in blue represent studies only containing isolate genomes. **b**, Rarefaction curves of the number of species detected as a function of the number of nonredundant genomes analyzed. Curves are depicted both for all the UHGG species and after excluding singleton species (represented by only one genome). **c**, Number of nonredundant genomes detected per species (left) alongside the degree of geographic diversity (calculated with the Shannon diversity index; right). Only the 25 most represented species clusters are depicted. **d**, Left, proportion of metagenomic reads from 1,005 independent datasets classified with Kraken 2 against the UHGG species representatives. Right, the degree of classification improvement provided over the standard Kraken 2 RefSeq database. The following correspond to the number of datasets analyzed per country: Cameroon, *n* = 54; Ethiopia, *n* = 25; Germany, *n* = 56; Ghana, *n* = 40; India, *n* = 105; Italy, *n* = 50; Luxembourg, *n* = 26; Russia, *n* = 4; Tanzania, *n* = 61; United Kingdom, *n* = 210; United States, *n* = 374. Box lengths represent the IQR of the data, and whiskers extend to the lowest and highest values within 1.5 times the IQR from the first and third quartiles, respectively.

**Extended Data Fig. 3:**
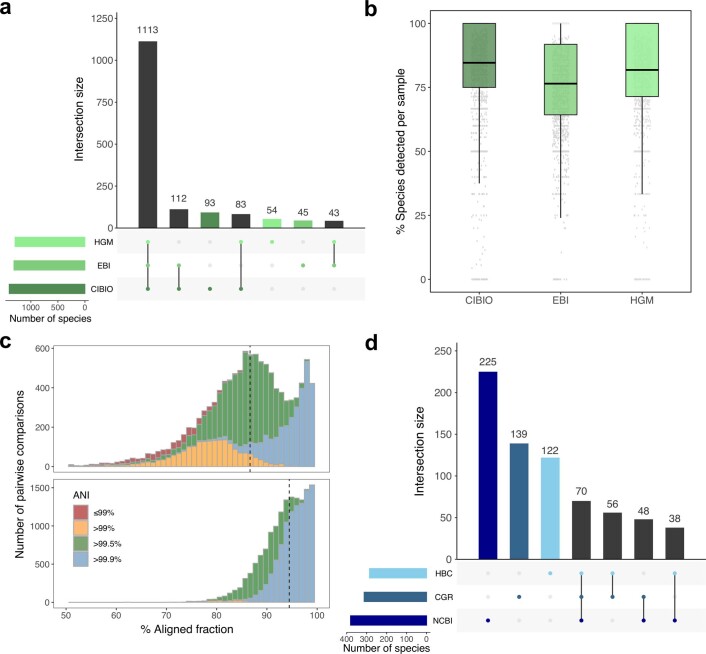
Species overlap across study sets. **a**, Number of species found across the three metagenome-assembled genome sets, ordered by their level of overlap. Only those genomes recovered from the 1,554 metagenomic samples used by all three studies were considered in this analysis. **b**, Distribution of the proportion of species recovered per sample (*n* = 1,554) in each study set out of all species recovered across all three studies in the same samples. Box lengths represent the IQR of the data, and the whiskers the lowest and highest values within 1.5 times the IQR from the first and third quartiles, respectively. **c**, Estimated aligned fractions and average nucleotide identities (ANI) between conspecific genomes obtained in the same sample but in different MAG studies. Results for medium-quality genomes are illustrated in the top panel, whereas those for near complete (≥90% completeness) genomes are represented in the lower panel. Vertical dashed lines denote the median values. **d**, Number of species identified in three culture-based studies and their degree of overlap. The NCBI study set consists mainly of genomes from the Human Microbiome Project (HMP).

We investigated how many of the 4,644 gut species were found in the different study collections to determine their level of overlap and reproducibility, as well as the ratio between cultured and uncultured species (Fig. 2a). Each of the large MAG studies used a different assembly and binning approach: the CIBIO study used metaSPAdes^31^ and MetaBAT 2 (ref. ^32^) for assembling and binning sequencing runs previously merged by sample; the HGM study used MEGAHIT^33^ to assemble runs merged by sample and applied a combination of MaxBin 2 (ref. ^34^), MetaBAT 2 (ref. ^32^), CONCOCT^35^ and DAS Tool^36^ for binning and refinement; and the EBI study used metaSPAdes^31^ and MetaBAT 2 (ref. ^32^) for assembling and binning individual runs without merging by sample. Despite these methodological differences, the largest intersection found was between these collections of MAGs, with the same 1,081 species detected independently in the CIBIO, EBI and HGM datasets, but not in any of the cultured genome studies. By restricting the analysis to genomes recovered from 1,554 samples common to all three MAG studies, we found that 93–97% of the species from each set were detected in at least one other MAG collection and 79–86% were detected across all three (Extended Data Fig. 3a). A similar level of species overlap was observed when comparing studies on a per-sample basis (Extended Data Fig. 3b). Furthermore, conspecific genomes recovered from the same samples across different studies had a median ANI and AF of 99.9% and 92.1%, respectively (94.5% AF with ≥90% complete genomes and 86.6% AF with medium-quality genomes; Extended Data Fig. 3c). These results suggest that the large-scale studies of human gut MAGs^16,18,20^ generally recovered highly similar genomes. However, the smaller AF values detected among genomes that were <90% complete suggest that caution is needed when using medium-quality genomes in downstream analyses.

Rarefaction analysis indicated that the number of uncultured species detected has not reached a saturation point, meaning that additional species remain to be discovered (Fig. 2b). However, these most likely represent rarer members of the human gut microbiome, as the number of species is closer to saturating when only considering those with at least two conspecific genomes.

We also investigated the intersection between the three large culture-based datasets: the HBC, CGR and NCBI (which contains gut genomes from the HMP^4^). Unlike the MAGs, the majority of cultured species were unique within a single collection (486/698; 70%), with only 70 (10%) common to all three collections (Extended Data Fig. 3d). This may be due to varied geographic sampling between the collections (Asia, Europe and North America) or might highlight the stochastic nature of culture-based studies.

### Most gut microbial species lack isolate genomes

We found that 3,750 (81%) of the species in the UHGG catalog did not have a representative in any of the human gut culture databases. To extend the search to isolate genomes from other environments or lacking information on the isolation source, we compared the UHGG catalog to all NCBI RefSeq isolate genomes. We identified an additional set of 438 species closely matching cultured genomes (88 from human body sites, 29 from other animals, 3 from plants and the remainder (318) from unknown sources), leaving 3,312 (71%) UHGG species as uncultured (Supplementary Table 2).

**Extended Data Fig. 4:**
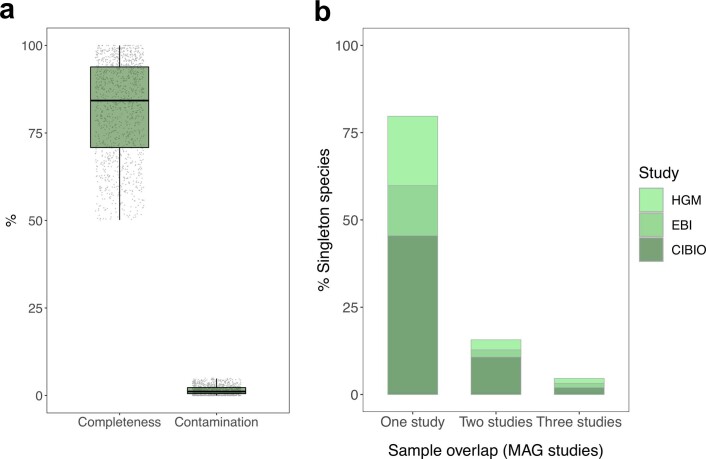
Quality and sample origin of uncultured singleton species. **a**, Genome completeness and contamination estimates of the 1,212 uncultured species represented by a single genome. Box lengths represent the IQR of the data, and the whiskers the lowest and highest values within 1.5 times the IQR from the first and third quartiles, respectively. **b**, Proportion of the 1,212 singleton species, by study set, that originated from samples analysed in one, two or three of the MAG studies (CIBIO, EBI and HGM).

**Extended Data Fig. 5:**
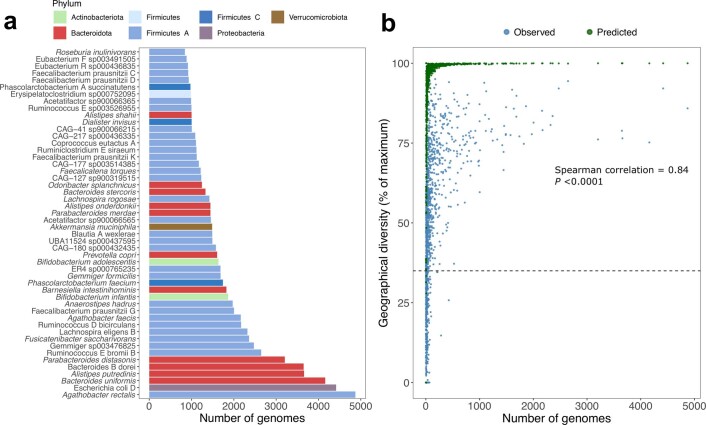
Species frequency and geographical diversity. **a**, Number of nonredundant genomes retrieved from the 50 most highly represented species in the UHGG catalog. Each species is colored by its assigned phylum according to the figure legend. **b**, Geographical diversity estimated using the Shannon index in relation to the number of nonredundant genomes from each species containing more than one genome (*n* = 2,786). Percentage values represent the estimated diversity normalized by the maximum theoretical value (considering an equal distribution of samples across the six major continents — Africa, Asia, Europe, North America, South America and Oceania). The Spearman’s rank correlation coefficient and *P* value (calculated with the Spearman’s test) are depicted in the graph. Predicted values represent the random geographical distribution of equivalent numbers of genomes observed for each species. Dashed horizontal line indicates the median observed value for species with more than one genome.

**Extended Data Fig. 6:**
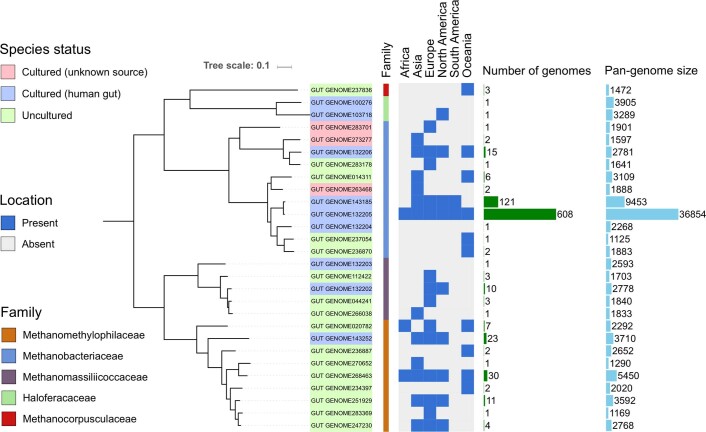
Diversity of the gut archaeal species detected. Phylogenetic tree of the 28 archaeal species detected in the human gut. Tips are labelled with the corresponding species representative code and colored according to its cultured status. The taxonomic affiliation (family), geographical distribution, number of nonredundant genomes and total pan-genome size are represented next to the tree.

By calculating the number of genomes contained within each cultured and uncultured human gut species, we found that species containing isolate genomes represented the largest clusters, while those exclusively encompassing MAGs tended to be the rarest, as discussed previously^16,18,20^. For example, only 2 of the 25 largest bacterial clusters were exclusively represented by MAGs (Fig. 2c), with 1,212 uncultured species represented by a single genome (80% of which originated from samples only analyzed in one of the MAG studies; Extended Data Fig. 4). The bacterial species most represented in our collection were *Agathobacter rectalis* (recently reclassified from *Eubacterium rectale*^37^), *Escherichia coli* D and *Bacteroides uniformis* (Fig. 2c, Extended Data Fig. 5a and Supplementary Table 2), whereas the most frequently recovered archaeal species was *Methanobrevibacter* A *smithii*, with 608 genomes found across all six continents (Extended Data Fig. 6). We inferred the level of geographic diversity of each species by calculating the Shannon diversity index on the proportion of samples in which each species was found per continent. The largest species clusters displayed similarly high levels of geographic distribution, indicating that the most highly represented species were not restricted to individual locations (Fig. 2c and Extended Data Fig. 5b).

We determined how representative the UHGG catalog is of the human gut microbial diversity by mapping 1,005 independent metagenomic datasets against the 4,644 UHGG species (Fig. 2d and Supplementary Table 4). Using Kraken 2 (ref. ^38^), we obtained a median classification rate of 85.9% (IQR = 83.5–88.1%). Notably, this corresponded to a median improvement of 155% over the standard RefSeq database. The increase in classification rate was most pronounced in non-Western samples from Cameroon, Ethiopia, Ghana and Tanzania, highlighting the potential of the UHGG catalog to improve the study of microbiome diversity from these understudied populations.

**Fig. 3:**
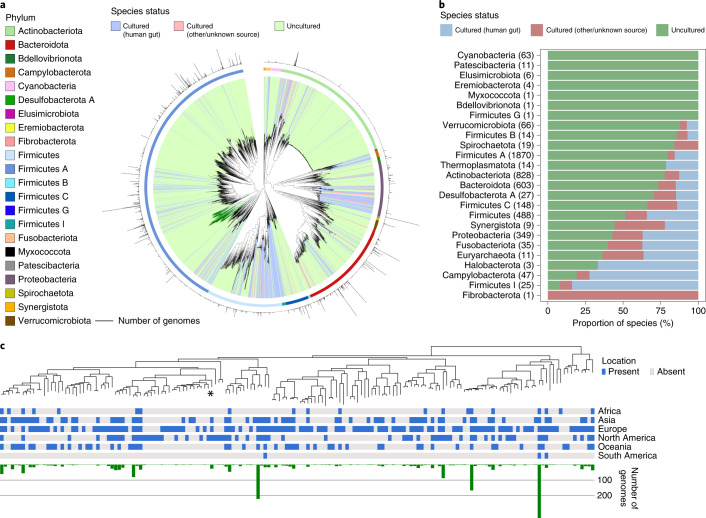
Uncultured species are predominant among human gut phyla. **a**, Maximum-likelihood phylogenetic tree of the 4,616 bacterial species detected in the human gut. Clades are colored by the cultured status of species, with outer circles depicting the GTDB phylum annotation. Bar graphs in the outermost layer indicate the number of genomes from each species. The order Comantemales ord. nov. is highlighted with dark green branches. **b**, Proportion of species within the 25 prokaryotic phyla detected according to cultured status. Numbers in parentheses represent the total number of species in the corresponding phylum. **c**, Phylogenetic tree of species belonging to the order Comantemales ord. nov. (phylum Firmicutes A), the largest phylogenetic group exclusively represented by uncultured species. The geographic distribution of each species and the number of genomes recovered are represented below the tree. The species previously classified as *Candidatus* ‘*Borkfalki ceftriaxensis*’ is indicated with an asterisk.

The phylogenetic distribution of the 4,616 bacterial (Fig. 3a) and 28 archaeal (Extended Data Fig. 6) species revealed that uncultured species exclusively represented 66% and 31% of the phylogenetic diversity of Bacteria and Archaea, respectively, with several phyla lacking cultured representatives (Fig. 3b). The four largest monophyletic groups lacking cultured genomes were the 4C28d-15 order (167 species, recently proposed as the novel order Comantemales ord. nov.^39^; Fig. 3c), order RF39 (139 species), family CAG-272 (88 species) and order Gastranaerophilales (67 species). While none have been successfully cultured, several have been described in the literature, including for RF39 (ref. ^16^) and Gastranaerophilales (previously classified as a lineage in the Melainabacteria^40^), which are characterized by highly reduced genomes with numerous auxotrophies. This analysis suggests that, despite recent culture-based studies^11,12,19,21^, much of the diversity in the gut microbiome remains uncultured, including several large and prevalent clades.

### Expanding the set of proteins in the human gut microbiome

**Fig. 4:**
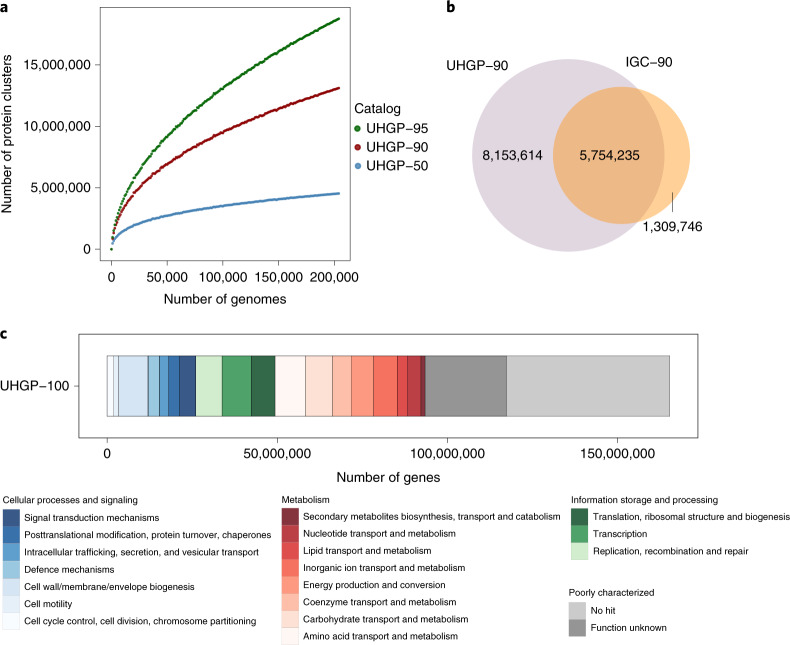
The UHGP improves coverage of the human gut protein landscape. **a**, Rarefaction curves of the number of protein clusters obtained as a function of the number of nonredundant genomes analyzed. Separate colored curves are depicted for the UHGP-95, UHGP-90 and UHGP-50. **b**, Overlap between the UHGP (purple) and IGC (orange), both clustered at 90% amino acid identity. **c**, COG functional annotation results of the unified gastrointestinal protein catalog clustered at 100% amino acid identity (UHGP-100).

**Extended Data Fig. 7:**
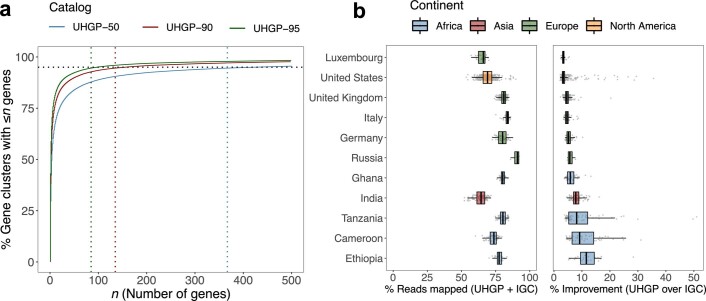
UHGP cluster size and mapping rate. **a**, Cumulative distribution curve of the number and size of the gene clusters of the UHGP-95 (*n* = 20,239,340), UHGP-90 (*n* = 13,907,849) and UHGP-50 (*n* = 4,735,546). Dashed vertical lines indicate the cluster size below which 90% of the gene clusters can be found. b, Proportion of metagenomic reads from 1,005 independent datasets aligned with DIAMOND against the combined clusters of UHGP-90 and IGC-90 (left). The degree of classification improvement provided over the IGC-90 alone is represented in the right panel. The following represents the number of datasets analysed per country: Cameroon, *n* = 54; Ethiopia, *n* = 25; Germany, *n* = 56; Ghana, *n* = 40; India, *n* = 105; Italy, *n* = 50; Luxembourg, *n* = 26; Russia, *n* = 4; Tanzania, *n* = 61; United Kingdom, *n* = 210; United States, *n* = 374. Box lengths represent the interquartile range (IQR) of the data, and the whiskers the lowest and highest values within 1.5 times the IQR from the first and third quartiles, respectively.

Metagenomic approaches have the ability to leverage gene content information not only for more precise taxonomic analysis but also to predict the functional capacity of individual species of interest in comparison to marker-gene-based methods (for example, relying solely on the 16S rRNA gene or a limited number of diagnostic genes). We built the UHGP catalog with a total of 625,255,473 full-length protein sequences predicted from the 286,997 analyzed genomes herein. These were clustered at 50% (UHGP-50), 90% (UHGP-90), 95% (UHGP-90) and 100% (UHGP-100) amino acid identity, generating between 5 to 171 million protein clusters (Fig. 1c and Extended Data Fig. 7a). While the number of UHGP-95 and UHGP-90 clusters showed a steady increase as a function of the number of genomes considered, those from UHGP-50 are reaching a saturation point (Fig. 4a), in line with previous estimates^6^.

To determine how comprehensive the UHGP is when compared to existing human gut gene catalogs, we combined the UHGP-90 (*n* = 13,910,025 protein clusters) with the IGC^5^, a collection of 9.9 million genes from 1,267 gut metagenome assemblies, which we grouped into 7,063,981 protein clusters at 90% protein identity (referred to as IGC-90). Nearly all samples used to generate the IGC were also included in the UHGP catalog (except for 59 transcriptome datasets), but the latter was generated from a larger and more geographically diverse metagenomic dataset (including samples from Africa, South America and Oceania). Combining the UHGP-90 and IGC-90 resulted in a set of 15.2 million protein clusters, with an overlap of 5.8 million sequences (Fig. 4b). This revealed that 81% of the IGC is represented in the UHGP catalog, with the missing 19% likely representing fragments of prokaryotic genomes that are <50% complete and viral or eukaryotic sequences, plasmids or other sequences not binned into MAGs. In fact, only 0.2% (*n* = 34,070 clusters) of the UHGP-90 was predicted to be of viral origin (on the basis of eggNOG annotations), as compared to the 5% estimate obtained in a previous human gut gene catalog^6^ included in the IGC. Most notably, though, the UHGP provided an increase of 115% in coverage of the gut microbiome protein space over the IGC (from 7,063,981 to a total of 15,217,595 protein clusters).

We also compared the read mapping rate using the same 1,005 metagenomic samples tested against the UHGG catalog (Supplementary Table 4). Even though the classification rate was substantially higher when using the UHGG catalog than with RefSeq, the increase with the UHGP-90 over IGC-90 was more modest (median of 5%; Extended Data Fig. 7b). These results suggest that, although the UHGP collectively encompasses a much larger number of protein clusters, most of the newly added proteins are at lower abundance/prevalence within individual samples. However, as the UHGP was generated from individual genomes and not from their original unbinned metagenome assemblies, our catalog also has the advantage of providing a direct link between each gene cluster and its genome of origin. To this end, we have also generated high-quality subsets of the UHGP-95, UHGP-90 and UHGP-50 consisting of protein clusters where at least two proteins from different genomes belonging to the same species were retrieved (UHGP-95-HQ, *n* = 10,798,224; UHGP-90-HQ, *n* = 8,082,122; UHGP-50-HQ, *n* = 3,088,278). This clustering criterion was used to control for the presence of contaminating sequences within each MAG and for the possibility that multiple copies of the same protein-coding sequence may be present in one genome. The UHGP ultimately allows the combination of individual genes with their genomic context for an integrated study of the gut microbiome.

### Functional capacity of the human gut microbiota

We used the eggNOG^41^, InterPro^42^, COG^43^ and KEGG^44^ annotation schemes to capture the full breadth of functions within the UHGP. However, we found that 41.5% of UHGP-100 was poorly characterized, as 27.3% lacked a match to any database and a further 14.2% only had a match to a COG with no known function (Fig. 4c). On the basis of the distribution of COG functions, the most highly represented categories were related to amino acid transport and metabolism, cell wall/membrane/envelope biogenesis and transcription.

**Extended Data Fig. 8:**
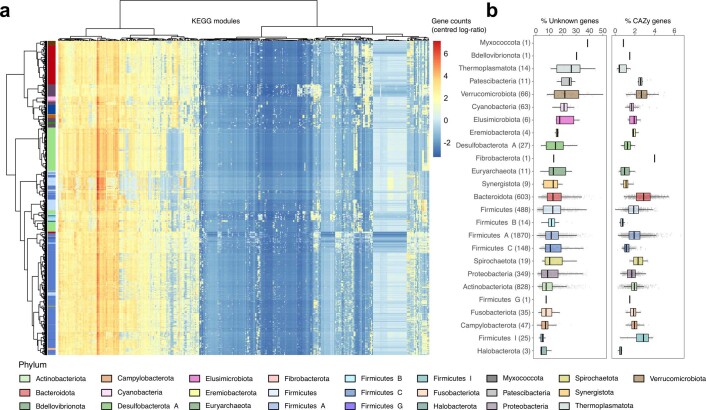
Functional annotation of gut microbiome species. **a**, Functional profiles of the UHGG species pan-genomes (rows) according to 363 KEGG modules (columns). Numbers of genes matching each module were normalized to centered log ratios after imputing values with zero counts. Species are colored according to phylum. KEGG modules and species were hierarchically clustered using the Ward’s criterion method. **b**, Proportion of each species pan-genome, partitioned by phylum, without any assignment to the eggNOG, InterPro, COG or KEGG databases (left). Proportion of the pan-genome with a match to the carbohydrate-active enzymes (CAZy) database (right). Sample size (number of species) of each phylum is indicated in parentheses (*n* = 4,644 total species). Box lengths represent the IQR of the data, and the whiskers the lowest and highest values within 1.5 times the IQR from the first and third quartiles, respectively.

We further leveraged the set of 171 million proteins derived from the human gut genomes to explore the functional diversity within each of the UHGG species. Protein sequences from all conspecific genomes were clustered at 90% amino acid identity to generate a pan-genome for each species. Analysis of the functional capacity of the UHGG species pan-genomes identified a total of 363 KEGG modules encoded by at least one species (Extended Data Fig. 8a and Supplementary Table 5). Most conserved modules were related to ribosomal structure, glycolysis, inosine monophosphate biosynthesis, gluconeogenesis and the shikimate pathway—all representing essential bacterial functions. However, we found that, for certain phyla such as Myxococcota, Bdellovibrionota, Thermoplasmatota, Patescibacteria and Verrucomicrobiota, a substantial proportion of the species pan-genomes remained poorly characterized (Extended Data Fig. 8b). At the same time, species belonging to the clades Fibrobacterota, Bacteroidota, Firmicutes I, Verrucomicrobiota and Patescibacteria had the highest proportion of genes encoding carbohydrate-active enzymes (CAZy; Extended Data Fig. 8b). As most of these lineages are largely represented by uncultured species (Fig. 3b), this suggests that the gut microbiota may harbor many species with important metabolic activities yet to be cultured and functionally characterized under laboratory conditions.

### Patterns of intraspecies genomic diversity

**Fig. 5:**
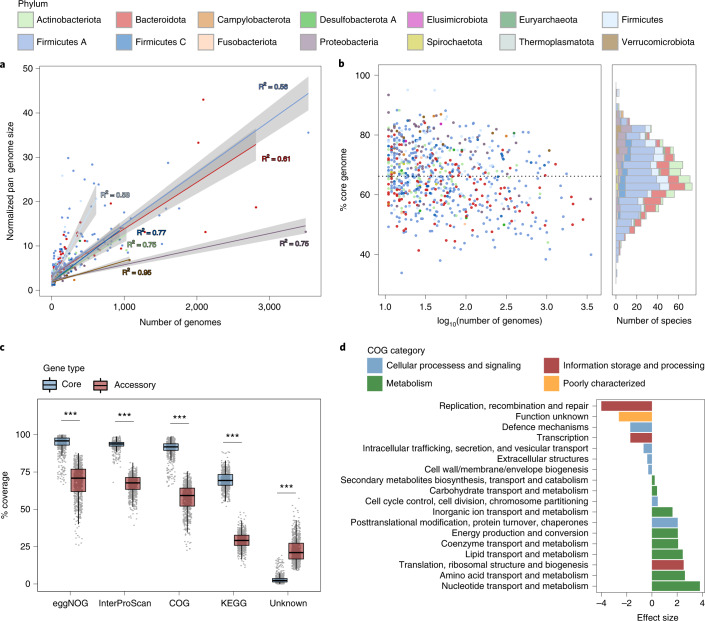
Pan-genome diversity patterns within the gut microbiome. **a**, Normalized pan-genome size as a function of the number of conspecific genomes. Regression curves were generated for each phylum, with the corresponding coefficients of determination indicated next to each curve and the shaded regions representing the 95% confidence level intervals. The following correspond to the number of species considered for each phylum: Actinobacteriota, *n* = 66; Bacteroidota, *n* = 122; Firmicutes, *n* = 90; Firmicutes A, *n* = 325; Firmicutes C, *n* = 44; Proteobacteria, *n* = 65; Verrucomicrobiota, *n* = 13. **b**, Fraction of the core genome for each species according to the number of conspecific genomes (left) and as a histogram (right), colored by phylum. The horizontal dashed line represents the median value across all species. **c**, Proportion of core and accessory genes (*n* = 781 species) classified with various annotation schemes, alongside the percentage of genes lacking any functional annotation. Box lengths represent the IQR of the data, and whiskers extend to the lowest and highest values within 1.5 times the IQR from the first and third quartiles, respectively. A two-tailed Wilcoxon rank-sum test was performed to compare the classification between the core and accessory genes (***P < 0.001). **d**, Comparison of the functional categories assigned to the core (*n* = 1,236,880) and accessory (*n* = 4,785,975) genes. Only statistically significant (adjusted *P* < 0.05) differences are shown. Significance was calculated with a two-tailed Wilcoxon rank-sum test and further adjusted for multiple comparisons using the Benjamini–Hochberg correction. A positive effect size (Cohen’s *d*) indicates over-representation in the core genes.

**Extended Data Fig. 9:**
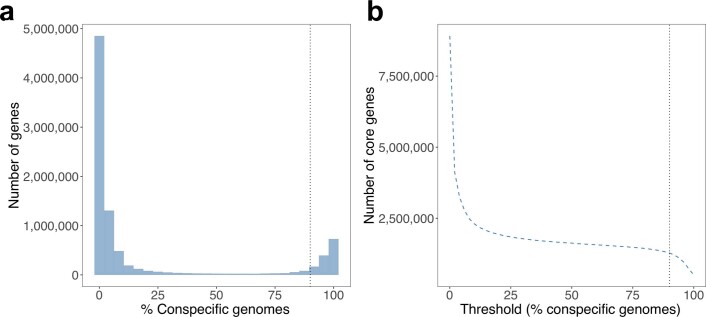
Gene frequency distribution within the species-level clusters. **a**, Distribution of the number of genes found per fraction of conspecific genomes. Only near-complete genomes (≥90% completeness) were considered in the analysis. **b**, Number of core genes detected based on the threshold of genomes per species used to classify as core. Vertical dashed line represents the 90% threshold used in this study.

With the protein annotations and pan-genomes inferred for each of the UHGG species, we explored their intraspecies core and accessory gene repertoire. Only near-complete genomes (≥90% completeness) and species with at least ten independent conspecific genomes were analyzed. The overall pattern of gene frequency within each of the 781 species considered here showed a distinctive bimodal distribution (Extended Data Fig. 9), with most genes classified as either core or rare (that is, present in ≥90% or <10% of conspecific genomes, respectively). We analyzed the pan-genome size for each species in relation to the number of conspecific genomes to look for differences in intraspecies gene richness. We observed distinct patterns across different gut phyla, with species from various Firmicutes clades showing the highest rates of gene gain (Fig. 5a). There was wide variation in the proportion of core genes between species even among clades with more than 1,000 genomes (Fig. 5b), with a median core genome proportion (percentage of core genes among all genes in the representative genome) estimated at 66% (IQR = 59.6–73.9%).

To distinguish the functions encoded in the core and accessory genes, we analyzed their associated annotations. Core genes were well covered, with a median of 96%, 94%, 92% and 69% of the genes assigned with an eggNOG, InterPro, COG and KEGG annotation, respectively (Fig. 5c). In contrast, the accessory genes had a significantly higher proportion of unknown functions (*P* < 0.001), with a median of 21% of the genes (IQR = 16.7–27.3%) lacking a match in any of the databases considered. Thereafter, we investigated the functions encoded by the core and accessory genes on the basis of the COG functional categories. Genes classified as core were significantly associated (adjusted *P* < 0.001) with key metabolic functions involved in nucleotide, amino acid and lipid metabolism, as well as other housekeeping functions (for example, related to translation and ribosomal structure; Fig. 5d). In contrast, accessory genes had a much greater proportion of COGs without a known function and of genes involved in replication and recombination, which are typically found in mobile genetic elements (MGEs; Fig. 5d). A significant number of accessory genes were related to defense mechanisms, which encompass not only general mechanisms of antimicrobial resistance (AMR) such as ABC transporter efflux pumps but also systems targeted toward invading MGEs (for example, CRISPR–Cas and restriction modification systems against bacteriophages). These results highlight the potential of this resource to provide better understanding of the dynamics of chromosomally encoded AMR within the gut and allow deciphering of the extent to which the microbiome may be a source of both known and novel resistance mechanisms.

**Fig. 6:**
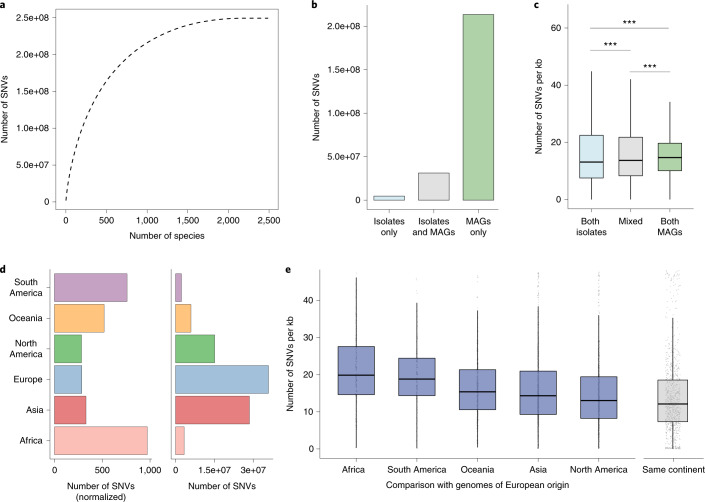
Analysis of intraspecies single-nucleotide variation. **a**, Total number of SNVs detected as a function of the number of species. The cumulative distribution was calculated after ordering the species by decreasing number of SNVs. **b**, Number of SNVs detected only in isolate genomes or MAGs, or in both. **c**, Pairwise SNV density analysis of genomes of the same or different type (isolates, *n* = 808,331 comparisons; mixed, *n* = 1,575,895 comparisons; MAGs, *n* = 26,899,457 comparisons). A two-tailed Wilcoxon rank-sum test was performed to assess statistical significance and further adjusted for multiple comparisons using the Benjamini–Hochberg correction (***P < 0.001). **d**, Left, the number of exclusive SNVs normalized by the number of genomes per continent. Right, the number of SNVs exclusively detected in genomes from each continent. **e**, Pairwise SNV density analysis between genomes from Europe, the largest genome subset, and other continents. The median SNV density was calculated per species, and the distribution is shown for all species (Africa, *n* = 188; Asia, *n* = 746; North America, *n* = 688; Oceania, *n* = 35; South America, *n* = 151). Comparison of genomes recovered from the same continent (*n* = 908 species) was used as a reference. The SNV density between genomes from the same continent is significantly lower (adjusted *P* < 0.05) than that calculated for genomes from different continents. In **c** and **e**, box lengths represent the IQR of the data, with whiskers depicting the lowest and highest values within 1.5 times the IQR of the first and third quartiles, respectively.

**Extended Data Fig. 10:**
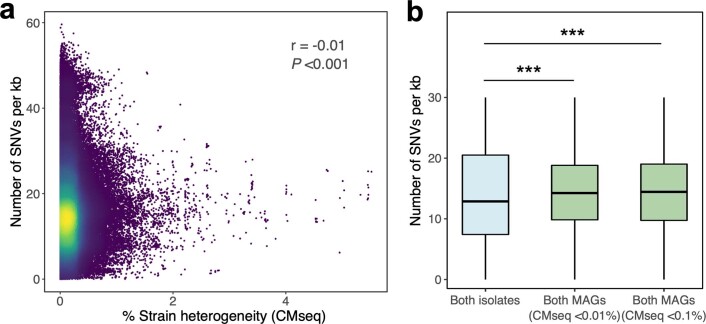
SNV density and MAG strain heterogeneity. **a**, Correlation between the SNV density calculated among MAGs and their level of strain heterogeneity estimated with CMseq (*n* = 268,994 comparisons). A Pearson correlation test was performed to determine the correlation coefficient and *P* value. Colors denote density of data points (increasing from dark purple to yellow). **b**, Comparison of pairwise SNV density between isolates (*n* = 808,331 comparisons) and between MAGs with <0.01% (*n* = 2,923,610 comparisons) and <0.1% strain heterogeneity (*n* = 13,634,222 comparisons). A two-tailed Wilcoxon rank-sum test was performed to assess statistical significance and further adjusted for multiple comparisons using the Benjamini-Hochberg correction (***P <0.001). Box lengths represent the IQR of the data, and the whiskers the lowest and highest values within 1.5 times the IQR from the first and third quartiles, respectively.

We next investigated intraspecies single-nucleotide variants (SNVs) within the UHGG species. We generated a catalog consisting of 249,435,699 SNVs from 2,489 species with three or more conspecific genomes (Fig. 6a). For context, a previously published catalog contained 10.3 million single-nucleotide polymorphisms from 101 gut microbiome species^45^. Of note, more than 85% of these SNVs were exclusively detected in MAGs, whereas only 2.2% were exclusive to isolate genomes (Fig. 6b). We found the overall pairwise SNV density between MAGs to be higher than that observed between isolate genomes (Fig. 6c). This was irrespective of the level of strain heterogeneity of the MAGs, as there was no correlation between SNV density and the degree of strain heterogeneity estimated with CMseq (Extended Data Fig. 10). Next, we assigned the detected SNVs to the continent of origin of each genome and observed that 36% of the SNVs were continent specific. Notably, genomes with a European origin contributed to the most exclusive SNVs (Fig. 6d). However, genomes from Africa contributed over three times more variation on average than European or North American genomes. Pairwise SNV analysis also supported a higher cross-continent SNV density, especially between genomes from Africa and Europe (Fig. 6e). Our results suggest that there is high strain variability between continents and that a considerable level of diversity remains to be discovered, especially from under-represented regions such as Africa, South America and Oceania.

### Resource implementation

Both the UHGG and UHGP catalogs are available as part of a new genome layer within the MGnify^46^ website, where summary statistics of each species cluster and their functional annotations can be interactively explored and downloaded (see ‘Data availability’ for more details). We have generated a Bitsliced Genomic Signature Index (BIGSI)^47^ of the UHGG catalog, which allows users to interactively query sequence fragments <5 kb in length to search for similar sequences in this collection.

We plan to periodically update the resource (approximately every 6–12 months) as new genomes are generated and made publicly available. MAGs will be retrieved from the European Nucleotide Archive (ENA), where a new MAG analysis class was recently implemented^48^. Genomes (MAGs or isolates) will be incorporated in the resource either as new species or by replacing uncultured reference genomes with better quality versions. We will adopt a versioning system whereby previous iterations of the catalog will still be accessible after subsequent updates to ensure reproducibility.

## Discussion

We have generated a unified sequence catalog representing over 200,000 genomes and 171 million protein sequences of the human gut microbiome. Of the 4,644 species contained in the UHGG catalog, 71% lack a cultured representative, meaning that the majority of microbial diversity in the catalog remains to be experimentally characterized. During preparation of our manuscript, a new collection of almost 4,000 cultured genomes from 106 gut species was released^49^, which will be incorporated in future versions of the resource. As 96% of these genomes were reported to have a species representative in the culture collections included here, we do not anticipate that this dataset will provide a substantial increase in the number of species discovered. Nevertheless, our analyses suggest that additional uncultured species from the human gut microbiome are yet to be discovered, highlighting the importance and need for culture-based studies. Furthermore, given the sampling bias toward populations from China, Europe and the United States, we expect that many under-represented regions still contain substantial uncultured diversity.

By comparing recently published large datasets of uncultured genomes^16,18,20^, we were able to assess the reproducibility of the results from each study. We show that, despite the different assembly, binning and refinement procedures used in the three studies, almost all of the same species and similar strains were recovered independently when using a consistent sample set. Although these results increase confidence in the use of MAGs, new methods for metagenome assembly, binning and quality control continue to be developed to overcome existing limitations, meaning that improved versions of the MAGs included here will likely be generated in the future.

With the establishment of this massive sequence catalog, it is evident that a large portion of the species and functional diversity within the human gut microbiome remains uncharacterized. Moreover, knowledge of the intraspecies diversity of many species is still limited owing to the presence of a small number of conspecific genomes. Having this combined resource can help guide future studies and prioritize targets for further experimental validation. Using the UHGG or UHGP catalogs, the community can now screen for the prevalence and abundance of species or genes in a large panel of intestinal samples and in specific clinical contexts. By pinpointing particular taxonomic groups with biomedical relevance, more targeted approaches could be developed to improve understanding of their role in the human gut. The functional predictions generated for the species pan-genomes could also be leveraged to develop new culturing strategies for isolation of candidate species. Target-enrichment methods such as single-cell^50^ and/or bait-capture hybridization^51^ approaches could also be applied. Given the large uncultured diversity still remaining in the human gut microbiome, having a high-quality catalog of all currently known species substantially enhances the resolution and accuracy of metagenome-based studies. Therefore, the presented genome and protein catalogs represent a key step toward a hypothesis-driven, mechanistic understanding of the human gut microbiome.

## Methods

### Genome collection

We compiled all the prokaryotic genomes publicly available as of March 2019 that were sampled from the human gut. To retrieve isolate genomes, we surveyed the IMG^24^, NCBI^22^ and PATRIC^23^ databases for genome sequences annotated as having been isolated from the human gastrointestinal tract. We complemented this set with bacterial genomes belonging to two recent culture collections: the HBC^19^ and CGR^21^. To avoid including duplicate entries due to redundancy between reference databases, we combined genomes obtained from the PATRIC and IMG repositories and added only those without an identical genome in the sets extracted from NCBI, HBC and CGR. This was determined by comparing isolate genomes between different databases using Mash v2.1 (ref. ^26^; ‘mash dist’ function) and only selecting one genome among those estimated to be identical (Mash distance of 0). MAGs (that is, uncultured genomes) were obtained from Pasolli et al.^20^ (CIBIO), Almeida et al.^18^ (EBI) and Nayfach et al.^16^ (HGM). For the CIBIO set, only genomes retrieved from samples collected from the intestinal tract were used.

Metadata for each genome were first retrieved from the five large human gut studies^16,18–21^. These were further enriched with data obtained using the ENA API (https://www.ebi.ac.uk/ena/portal/api) and the NCBI E-utilities (http://eutils.ncbi.nlm.nih.gov/). Metadata on the isolate genomes from IMG and PATRIC were retrieved using the GOLD^52^ system and the PATRIC FTP website (ftp://ftp.patricbrc.org/patric2/current_release/RELEASE_NOTES/genome_metadata), respectively. We only extracted metadata on the geographic origin of each genome, as other factors such as disease status and demographic information were missing from most of the samples.

### Assessing genome quality

Assembly statistics were calculated with the ‘stats.sh’ script from BBMap v38.75 (https://sourceforge.net/projects/bbmap/). Genome quality (completeness and contamination) was estimated with CheckM v1.0.11 (ref. ^53^) using the ‘lineage_wf’ workflow to select only genomes that passed the following criteria: >50% genome completeness, <5% contamination and an estimated quality score (completeness – 5 × contamination) > 50. We also searched for the presence of rRNAs in each genome with the ‘cmsearch’ function of INFERNAL v1.1.2 (ref. ^54^; options ‘-Z 1000 --hmmonly --cut_ga --noali --tblout’) against the Rfam^55^ covariance models for the 5S, 16S and 23S rRNAs. tRNAs of the standard 20 amino acids were identified with tRNAScan-SE v2.0 (ref. ^56^) with options ‘-A -Q’ for archaeal species and ‘-B -Q’ for species belonging to bacterial lineages.

To investigate the level of strain heterogeneity represented within each MAG, we used the CMseq tool (https://github.com/SegataLab/cmseq) as previously described^20^. Briefly, metagenomic reads from the sample used to generate the MAG were aligned to the respective MAG using bowtie v2.2.3 (ref. ^57^), with the resulting alignment file indexed and sorted with samtools v1.5 (ref. ^58^). The level of strain heterogeneity was estimated with the ‘polymut.py’ script from the CMseq package by calculating the number of nonsynonymous substitutions detected out of all positions mapped with a depth of coverage of at least 10 reads and base quality of at least 30 (a minimum of 100 positions were needed to estimate strain heterogeneity).

### Species clustering

We clustered the total set of 286,997 genomes at an estimated species level (ANI ≥ 95%; ref. ^27^) using dRep v2.2.4 (ref. ^59^) with the following options: ‘-pa 0.9 -sa 0.95 -nc 0.30 -cm larger’. Because of the computational burden of clustering the entire genome set, we used an iterative approach where random chunks of 50,000 genomes were clustered independently. The selected representatives from each chunk were combined and subsequently clustered, reducing the final computational load. To ensure that the best quality genome was selected as the species representative in each iteration, a score was calculated for each genome on the basis of the following formula:

$${\mathrm{Score}} = {\mathrm{CMP}} - 5 \times {\mathrm{CNT}} + 0.5 \times {\mathrm{log}}\left( {{\mathrm{N}}50} \right)$$

where CMP represents the completeness level, CNT is the estimated contamination and N50 is the assembly contiguity characterized by the minimum contig size in which half of the total genome sequence is contained. The genome with the highest score was chosen as the species representative, with cultured genomes prioritized over uncultured genomes (that is, if a MAG had a higher score than an isolate genome, the latter would still be chosen as the representative).

To further investigate the within-species population diversity, we calculated pairwise distances for all conspecific genomes using Mash v2.1 (ref. ^26^; default sketch size). From these results, we generated individual distance trees for each species using the ‘complete’ hierarchical clustering method implemented in the Fastcluster R package^60^. We calculated the number of clusters recovered using a distance cutoff of 0.03 (97% ANI) and 0.01 (99% ANI).

### Evaluating reproducibility of the methods

The species clusters inferred here were compared with those previously generated in human gut MAG studies^16,18,20^ from a common set of genomes. Similarity between species clusterings was estimated using the adjusted Rand index (ARI) computed in the Scikit-learn Python package^61^. This metric considers both the number of clusters and cluster membership to compute a similarity score ranging from 0 to 1.

Conspecific genomes recovered in the same metagenomic samples but in different studies were compared with FastANI v1.1 (ref. ^27^) with default parameters to obtain both the maximum AF and ANI for each pairwise comparison.

### Inferring cultured status

To determine cultured status, the UHGG species representatives were searched against NCBI RefSeq release 93 after excluding uncultured genomes (that is, metagenome-assembled or single-cell amplified genomes). Genome alignments were performed in two stages: (1) Mash v2.1 (ref. ^26^) was used as an initial screen (using the function ‘mash dist’) to identify the most similar RefSeq genome to each of the UHGG species and (2) ‘dnadiff’ from MUMmer v4.0.0beta2 (ref. ^62^) was subsequently used to compute whole-genome ANI for the genome pairs. A species was considered to have been cultured if (1) it contained a cultured gut genome from the UHGG catalog or (2) it matched an isolate RefSeq genome with at least 95% ANI over at least 30% of the genome length. Available metadata related to each RefSeq genome were retrieved from the ENA API (https://www.ebi.ac.uk/ena/portal/api/) using the corresponding BioSample accession.

### Calculating the number of conspecific genomes

For an accurate assessment of the number of nonredundant genomes belonging to each species, we de-replicated all conspecific genomes at a 99.9% ANI threshold using dRep with options ‘-pa 0.999 --SkipSecondary’. Furthermore, the frequency of each species was only counted once per sample to avoid cases where the same genome was recovered multiple times because of overlapping samples between the three MAG studies.

### Estimating geographic diversity

A geographic diversity index was estimated to assess how widely distributed each species was. We calculated the Shannon diversity index on the proportion of samples in which each species was found per continent. This metric combines both richness and evenness, such that the level of estimated diversity is highest in species found across all continents at a similar proportion.

### Metagenomic read mapping

A set of 1,005 metagenomic datasets from 14 studies (Supplementary Table 4) were retrieved from ENA and used to perform read mapping against the genome (UHGG) and protein (UHGP) catalogs. Only studies that were not used to generate the UHGG or UHGP catalogs were included. Reads were quality filtered and trimmed using TrimGalore v0.6.0 (https://github.com/FelixKrueger/TrimGalore), and human contamination was removed by aligning the reads with BWA MEM v0.7.16a-r1181 (ref. ^63^; default options) against human genome GRCh38. Filtered reads were then mapped using Kraken v2.0.8-beta^38^ (with default settings) against a custom database of the UHGG catalog available from the MGnify^46^ FTP site (http://ftp.ebi.ac.uk/pub/databases/metagenomics/mgnify_genomes/), and the standard RefSeq database (release 96). Bracken^64^ databases of the UHGG catalog for read lengths of 50, 100, 150, 200 and 250 bp were also generated and have been made available from the MGnify FTP site. Classification improvement was calculated on a per-sample basis as (proportion of reads classified with UHGG − proportion of reads classified with RefSeq)/proportion of reads classified with RefSeq × 100. DIAMOND v0.9.21.122 (ref. ^65^) was used to translate and map the reads against the IGC-90 and UHGP-90 protein catalogs using the ‘blastx’ function with options ‘--id 90 --evalue 1e-6 -k 1 --max-hsps 1’.

### Phylogenetic analyses

Taxonomic annotation of each species representative was performed with GTDB-Tk v0.3.1 (refs. ^29,30^; database release 04-RS89) using the ‘classify_wf’ function and default parameters. To use consistent species boundaries between the genome clustering and taxonomic classification procedures, genomes were assigned at the species level if the ANI to the closest GTDB-Tk species representative genome was ≥95% and the AF was ≥30%. In this taxonomy scheme, genera and species names with an alphabetic suffix indicate taxa that are polyphyletic or needed to be subdivided on the basis of taxonomic rank normalization according to the current GTDB reference tree. The lineage containing the type strain retains the unsuffixed (valid) name, and all other lineages are given alphabetic suffixes, indicating that they are placeholder names that need to be replaced in due course. Taxon names above the rank of genus appended with an alphabetic suffix indicate groups that are not monophyletic in the GTDB reference tree but for which there exists alternative evidence that they are monophyletic groups. We also generated NCBI taxonomy annotations for each species-level genome on the basis of its placement in the GTDB tree, using the ‘gtdb_to_ncbi_majority_vote.py’ script available in the GTDB-Tk repository (https://github.com/Ecogenomics/GTDBTk/).

Maximum-likelihood trees were generated de novo using the protein sequence alignments produced by GTDB-Tk: we used IQ-TREE v1.6.11 (ref. ^66^) to build a phylogenetic tree of the 4,616 bacterial and 28 archaeal species. The best fit model was automatically selected by ‘ModelFinder’ on the basis of the Bayesian information criterion (BIC) score. The LG+F+R10 model was chosen for building the bacterial tree, while the LG+F+R4 model was used for the archaeal phylogeny. Trees were visualized and annotated with Interactive Tree Of Life (iTOL) v4.4.2 (ref. ^67^). Phylogenetic diversity (PD) was estimated by the sum of branch lengths, with the amount that was exclusive to uncultured species calculated as PD_total_ – PD_cultured_. Uncultured monophyletic groups were defined as nodes in the tree containing child leaves exclusively comprising uncultured genomes.

### BIGSI construction

A BIGSI^47^ was generated for all species-level genomes with BIGSI v0.3.8. First, *k*-mers of size 31 were extracted from each genome with McCortex v1.0.1 (ref. ^68^; ‘mccortex31 build -k 31’). Thereafter, Bloom filters were built for each *k*-mer set using ‘bigsi bloom’ and inserted into the BIGSI index with ‘bigsi build’. BIGSI config parameters *h* (number of hash functions applied to each *k*-mer) and *m* (Bloom filter’s length in bits) were set at 1 and 28,000,000, respectively. A final API layer for querying the index was built using hug (http://www.hug.rest/) and hosted on the MGnify^46^ website at https://www.ebi.ac.uk/metagenomics/genomes.

### Pan-genome analysis and functional annotation

Protein-coding sequences (CDS) for each of the 286,997 genomes were predicted and annotated with Prokka v1.13.3 (ref. ^69^), using Prodigal v2.6.3 (ref. ^70^) with options ‘-c’ (predict proteins with closed ends only), ‘-m’ (prevent genes from being built across stretches of sequence marked as Ns) and ‘-p single’ (single mode for genome assemblies containing a single species). Pan-genome analyses were carried out using Roary v3.12.0 (ref. ^71^). We set a minimum amino acid identity for a positive match at 90% (‘-i 90’), a core gene defined at 90% presence (‘-cd 90’) and no paralog splitting (‘-s’). Normalized pan-genome size was estimated by dividing the total number of core and accessory genes by the number of genes contained in the species representative genome.

The UHGP catalog was generated from the combined set of 625,255,473 CDS predicted. Protein clustering of the UHGP and IGC^5^ was performed with the ‘linclust’ function of MMseqs2 v6-f5a1c^72^ with options ‘--cov-mode 1 -c 0.8’ (minimum coverage threshold of 80% the length of the shortest sequence) and ‘--kmer-per-seq 80’ (number of *k*-mers selected per sequence, increased from the default of 21 to improve clustering sensitivity). The ‘--min-seq-id’ option was set at 1, 0.95, 0.9 and 0.5 to generate the catalogs at 100%, 95%, 90% and 50% protein identity, respectively. We clustered the IGC only at 90% and 50% protein identity, as it was originally de-replicated at 95% nucleotide identity^5^. Functional characterization of all protein sequences was performed with eggNOG-mapper v2 (ref. ^73^; database v5.0 (ref. ^41^)) and InterProScan v5.35-74.0 (ref. ^42^). COG^43^, KEGG^44^, CAZy^74^ and viral annotations were derived from the eggNOG-mapper results. Differences in annotation coverage and COG functional categories between the core and accessory genes were evaluated with two-tailed Wilcoxon rank-sum tests in R v3.6.0 (function ‘wilcox.test’). Expected *P* values were corrected for multiple testing with the Benjamini–Hochberg method. Cohen’s *d* effect sizes were estimated with the function ‘cohen.d’ from the Effsize^75^ R package. To accurately estimate the proportion of each KEGG module in the species pan-genome, we used the compositional data analysis R package CoDaSeq^76^. Pseudocounts for zero-count data were first imputed using a ﻿Bayesian–multiplicative simple replacement procedure implemented in the ‘cmultRepl’ function (method ‘CZM’). Final counts were thereby converted to centered log ratios using the ‘codaSeq.clr’ function to account for the compositional nature of the data and for differences in pan-genome size.

### SNV analyses

A total of 2,489 species with at least three conspecific genomes were used to generate a catalog of SNVs. For each species, we mapped all conspecific genomes to the representative genome using the ‘nucmer’ program from MUMmer v4.0.0.beta2 (ref. ^62^) and filtered alignments using the ‘delta-filter’ program with options ‘-q -r’ to exclude chance- and repeat-induced alignments. Thereafter, we identified SNVs using the ‘show-snps’ program. Single-base insertions and deletions were not counted as SNVs. Each SNV locus was included in the catalog only when the alternate allele was detected in at least two conspecific genomes. The final SNV catalog was generated by unifying the SNV coordinates on the basis of their position in the species representative genome. The SNV entries in the catalog were characterized as genome type or continent specific on the basis of whether the alternate allele could be found solely in genomes from a specific genome type or continent. The number of continent-specific SNVs was normalized by the number of genomes from the corresponding continent to estimate the contribution per genome to the continent-specific SNV discoveries.

Similar programs and parameters were used for the pairwise genome alignment, but in this case only near-complete genomes (≥90% completeness) and species with at least ten independent conspecific genomes were considered. Because of the high computational demand, pairwise alignments of species encompassing more than 1,000 genomes were limited to the 1,000 best quality genomes. A total of 29,283,684 pairwise genome alignments were performed between almost 113,000 genomes from 909 species. For each pairwise comparison, we estimated the total number of SNVs and the overall density as the number of SNVs per kilobase. In addition, the pairwise comparisons were organized on the basis of the type and continent origin of the genomes in the pair for further downstream analyses. A two-tailed Wilcoxon rank-sum test was used to evaluate differences in SNV distribution. Resulting *P* values were corrected for multiple testing with the Benjamini–Hochberg method.

### Reporting Summary

Further information on research design is available in the Nature Research Reporting Summary linked to this article.

## Online content

Any methods, additional references, Nature Research reporting summaries, source data, extended data, supplementary information, acknowledgements, peer review information; details of author contributions and competing interests; and statements of data and code availability are available at 10.1038/s41587-020-0603-3.

## Supplementary information

Reporting Summary

Supplementary Table 1 Assembly statistics and metadata of the 286,997 human gut microbial genomes used to generate the UHGG collection.

Supplementary Table 2 General statistics and pan-genome results of the 4,644 representative species within the UHGG catalog.

Supplementary Table 3 Species clustering similarity between the UHGG catalog and the original human gut MAG studies.

Supplementary Table 4 Human gut metagenomic datasets mapped against the genome and protein catalogs.

Supplementary Table 5 Number of genes from each of the 4,644 UHGG species assigned to 363 KEGG modules.

## Data Availability

Genome assemblies of the UHGG catalog have been deposited in the European Nucleotide Archive under study accession ERP116715. The UHGG, UHGP and SNV catalogs are available from the MGnify FTP site (http://ftp.ebi.ac.uk/pub/databases/metagenomics/mgnify_genomes/) alongside functional annotations, pan-genome results and custom Kraken 2/Bracken databases of the UHGG catalog. These data, together with the BIGSI search index of the UHGG catalog, can also be accessed interactively via the MGnify website at https://www.ebi.ac.uk/metagenomics/genomes. Mash distance trees have been generated for each individual species cluster and are available at both the MGnify website and the associated FTP site.

## Code availability

The workflow used to generate the genome and protein catalogs, alongside the pan-genome and functional annotations, is described in a Common Workflow Language (CWL) pipeline at https://github.com/EBI-Metagenomics/genomes-pipeline. Scripts used to generate the SNV catalogs are available at https://github.com/zjshi/snv_analysis_almeida2019.
