# Supplementary material for: A unified catalog of 204,938 reference genomes from the human gut microbiome
Source: Nat Biotechnol. 2020 Jul 20;39(1):105–14. doi: 10.1038/s41587-020-0603-3 (PMC7801254; doi:10.1038/s41587-020-0603-3)
Supplement: Supplementary file 1 — Reporting Summary [file 41587_2020_603_MOESM1_ESM.pdf]

nature research

Corresponding author(s): Alexandre Almeida and Robert D. Finn

Last updated by author(s): May 21, 2020

Reporting Summary

Nature Research wishes to improve the reproducibility of the work that we publish. This form provides structure for consistency and transparency in reporting. For further information on Nature Research policies, see Authors & Referees and the Editorial Policy Checklist.

Statistics

For all statistical analyses, confirm that the following items are present in the figure legend, table legend, main text, or Methods section.

n/a	Confirmed	
☐	☒	The exact sample size (*n*) for each experimental group/condition, given as a discrete number and unit of measurement
☒	☐	A statement on whether measurements were taken from distinct samples or whether the same sample was measured repeatedly
☐	☒	The statistical test(s) used AND whether they are one- or two-sided<br>*Only common tests should be described solely by name; describe more complex techniques in the Methods section.*
☒	☐	A description of all covariates tested
☐	☒	A description of any assumptions or corrections, such as tests of normality and adjustment for multiple comparisons
☐	☒	A full description of the statistical parameters including central tendency (e.g. means) or other basic estimates (e.g. regression coefficient) AND variation (e.g. standard deviation) or associated estimates of uncertainty (e.g. confidence intervals)
☐	☒	For null hypothesis testing, the test statistic (e.g. *F*, *t*, *r*) with confidence intervals, effect sizes, degrees of freedom and *P* value noted<br>*Give P values as exact values whenever suitable.*
☒	☐	For Bayesian analysis, information on the choice of priors and Markov chain Monte Carlo settings
☒	☐	For hierarchical and complex designs, identification of the appropriate level for tests and full reporting of outcomes
☐	☒	Estimates of effect sizes (e.g. Cohen's *d*, Pearson's *r*), indicating how they were calculated

Our web collection on statistics for biologists contains articles on many of the points above.

Software and code

Policy information about availability of computer code

Data collection	European Nucleotide Archive (ENA); National Center for Biotechnology Information (NCBI); Integrated Microbial Genomes & Microbiomes (IMG/M); Pathosystems Resource Integration Center (PATRIC)
Data analysis	CheckM v1.0.11; INFERNAL v1.1.2; tRNAScan-SE v2.0; dRep v2.2.4; FastANI v1.1; Python v2.7 and v3.6; R v3.5; GTDB-Tk v0.3.1; Mash v2.1; MUMmer v4.0.0beta2; IQ-TREE v1.6.11; BIGSI v0.3.8; Prokka v1.13.3; Prodigal v2.6.3; Roary v3.12.0; MMseqs2 v6-f5a1c; eggNOG-mapper v2; InterProScan v5.35-74.0; Kraken v2.0.8-beta; Bracken v2.5;Trim Galore v0.6.0; BWA v0.7.16a-r1181; bowtie v2.2.3; samtools v1.5; DIAMOND v0.9.21.122; BBMap v38.75; CMseq (https://github.com/SegataLab/cmseq); iTOL v4.4.2; gtdb_to_ncbi_majority_vote.py (https://github.com/Ecogenomics/GTDBTk/).

For manuscripts utilizing custom algorithms or software that are central to the research but not yet described in published literature, software must be made available to editors/reviewers. We strongly encourage code deposition in a community repository (e.g. GitHub). See the Nature Research guidelines for submitting code & software for further information.

Data

Policy information about availability of data

All manuscripts must include a data availability statement. This statement should provide the following information, where applicable:

- Accession codes, unique identifiers, or web links for publicly available datasets
- A list of figures that have associated raw data
- A description of any restrictions on data availability

Genome assemblies of the UHGG have been deposited in the European Nucleotide Archive under study accession ERP116715. The UHGG, UHGP and SNV catalogs are available from the MGnify FTP site (http://ftp.ebi.ac.uk/pub/databases/metagenomics/mgnify_genomes/) alongside functional annotations, pan-genome results and custom Kraken 2/Bracken databases of the UHGG. These data together with the BIGSI search index of the UHGG can also be accessed interactively via the MGnify website: https://www.ebi.ac.uk/metagenomics/genomes. Mash distance trees have been generated for each individual species cluster and are available both in the MGnify website and the associated FTP site.

Field-specific reporting

Please select the one below that is the best fit for your research. If you are not sure, read the appropriate sections before making your selection.

☒ Life sciences ☐ Behavioural & social sciences ☐ Ecological, evolutionary & environmental sciences

For a reference copy of the document with all sections, see nature.com/documents/nr-reporting-summary-flat.pdf

Life sciences study design

All studies must disclose on these points even when the disclosure is negative.

Sample size	No sample size calculation was performed, as the aim was to include all publicly available human gut microbial genomes available (n = 286,997 genomes as of March 2019).
Data exclusions	Genomes were filtered to select those with an estimated completeness >50%, contamination <5% and quality score (completeness - 5*contamination) >50. Quality criteria for exclusion was pre-established based on existing literature and current standards in the field.
Replication	By comparing recently published large datasets of uncultured genomes, we were able to assess the reproducibility of the results from each study. We show that despite the different assembly, binning and refinement procedures employed in the three studies, almost all of the same species and similar strains were recovered independently when using a consistent sample set.
Randomization	Randomization was not relevant to this study, as we analysed publicly available data.
Blinding	Not relevant to this study, as we analysed publicly available data.

Reporting for specific materials, systems and methods

We require information from authors about some types of materials, experimental systems and methods used in many studies. Here, indicate whether each material, system or method listed is relevant to your study. If you are not sure if a list item applies to your research, read the appropriate section before selecting a response.

Materials & experimental systems

n/a	Involved in the study	
☒	☐	Antibodies
☒	☐	Eukaryotic cell lines
☒	☐	Palaeontology
☒	☐	Animals and other organisms
☒	☐	Human research participants
☒	☐	Clinical data

Methods

n/a	Involved in the study	
☒	☐	ChIP-seq
☒	☐	Flow cytometry
☒	☐	MRI-based neuroimaging
